# Supplementary material for: Effects of trapping effort and trap placement on estimating abundance of Humboldt’s flying squirrels
Source: PeerJ. 2019 Oct 3;7:e7783. doi: 10.7717/peerj.7783 (PMC6778666; doi:10.7717/peerj.7783)
Supplement: Table S1 — We present model structure, Akaike’s Information Criterion adjusted for sample size (AICC), change in AICC from the top-ranking model (ΔAICC), AICC weight of evidence (w), and the number of parameters (K). [file peerj-07-7783-s007.docx]

**Table S1:**

**Models used to determine the most-supported model structure for the emigration and immigration of Humboldt’s flying squirrels captured on 16 sites during 2 studies in Oregon, USA. We present model structure, Akaike’s Information Criterion adjusted for sample size (AIC_C_), change in AIC_C_ from the top-ranking model (ΔAIC_C_), AIC_C_ weight of evidence (w), and the number of parameters (K).**

| Model^a^ | AIC_C_ | ΔAIC_C_ | *w* | K |
| --- | --- | --- | --- | --- |
| ᵧ''(Null) = ᵧ'(Null) = 0 | 148125.15 | 0.00 | 0.68 | 123 |
| ᵧ''(Null) = ᵧ'(Null) | 148127.24 | 2.09 | 0.24 | 124 |
| ᵧ''(Null) ᵧ'(Null) | 148129.28 | 4.13 | 0.09 | 125 |
| ᵧ''(Null) ᵧ'(Site) | 148141.12 | 15.97 | 0.00 | 133 |
| ᵧ''(Site) ᵧ'(Null) | 148154.90 | 29.76 | 0.00 | 140 |
| ᵧ''(Site) = ᵧ'(Site) | 148158.76 | 33.61 | 0.00 | 139 |
| ᵧ''(Site) ᵧ'(Site) | 148171.46 | 46.31 | 0.00 | 148 |

^a^ Model structures for initial capture (*p*), recapture (*c*) probabilities were held to a site by trapping day model structure (Site*Time), and model structures for apparent annual survival were held to a site by year model structure (Site*Year).
