# Supplementary material for: Effects of trapping effort and trap placement on estimating abundance of Humboldt’s flying squirrels
Source: PeerJ. 2019 Oct 3;7:e7783. doi: 10.7717/peerj.7783 (PMC6778666; doi:10.7717/peerj.7783)
Supplement: Table S2 — We present models ranked based on support using Akaike’s Information Criterion adjusted for sample size (AICC), change in AICC from the top-ranking model (ΔAICC), AICC weight of evidence (w), and the number of parameters (K). [file peerj-07-7783-s008.docx]

**Table S2:**

**Models of trap-response behaviors for Humboldt’s flying squirrels captured on 16 sites during 2 studies in Oregon, USA. We present models ranked based on support using Akaike’s Information Criterion adjusted for sample size (AIC_C_), change in AIC_C_ from the top-ranking model (ΔAIC_C_), AIC_C_ weight of evidence (w), and the number of parameters (K).**

| Model^a^ | AIC_C_ | ΔAIC_C_ | *w* | K |
| --- | --- | --- | --- | --- |
| p(Time)c(Time) | 148467.01 | 0.00 | 1.00 | 63 |
| p(Null)c(Time) | 148495.00 | 27.98 | 0.00 | 62 |
| p(Time)c(Null) | 148528.08 | 61.06 | 0.00 | 62 |
| p(Null)c(Null) | 148556.06 | 89.05 | 0.00 | 61 |
| p(Time) = c(Time) | 148609.15 | 142.14 | 0.00 | 61 |
| p(Null) = c(Null) | 148619.16 | 152.15 | 0.00 | 60 |

^a^ Model structures for apparent annual survival were held to a site by year model structure (Site*Year), while model structures for emigration and immigration were fixed to zero.
